# Comparison Between Video Laryngoscope and Direct Laryngoscope for Pressor Response in Adult Patients Undergoing Craniotomy

**DOI:** 10.7759/cureus.91603

**Published:** 2025-09-04

**Authors:** Nikita Gupta, Deepti Saigal, Priyanka Gupta, Preeti Thakur

**Affiliations:** 1 Department of Anesthesia and Intensive Care, Vardhman Mahavir Medical College and Safdarjung Hospital, New Delhi, IND

**Keywords:** arterial pressure, intraocular pressure, intubation, laryngoscope, neurosurgery

## Abstract

Objectives: This prospective randomized study was done to compare hemodynamic and intraocular pressure (IOP) responses to laryngoscopy and intubation with the use of the Macintosh direct laryngoscope (DL) (Medisearch Systems Pvt. Ltd., Jalandhar, India) and C-MAC video laryngoscope (VL) (C-MAC®, Karl Storz, Tuttlingen, Germany) in patients undergoing brain tumor excision surgery.

Materials and methods: Sixty American Society of Anesthesiologists (ASA) grade I and II patients aged 18-65 years undergoing fronto-parietal brain tumor excision surgery were randomly allocated into two groups: group DL (intubation using Mcintosh DL) (n=30) and group VL (intubation with C-MAC® VL) (n=30). Baseline values of mean arterial pressure (MAP), systolic blood pressure (SBP), diastolic blood pressure (DBP), heart rate (HR), and IOP were recorded. The primary outcome was the percentage change in MAP. Secondary outcomes were percentage changes in HR, SBP, DBP, and IOP. Hemodynamic parameters were noted during laryngoscopy (T_L1_, T_L2_, T_L3_) and every 30 seconds after intubation (T_1_-T_10_). IOP was measured every minute for five minutes post-intubation. Blood pressures were measured using invasive arterial blood pressure, and IOP was measured using Schiotz tonometry.

Statistical analysis: Categorical variables were presented in numbers and percentages, and continuous variables were presented as mean±SD and median. Normality of data was tested by the Kolmogorov-Smirnov test. The unpaired t-test/Mann-Whitney U test was used for comparing quantitative variables, and the chi-squared test/Fisher's exact test was used for qualitative variables. A p-value lower than 0.05 was considered statistically significant.

Results: The percentage rise in MAP was significantly greater in the DL group as compared with the VL group at all time points during laryngoscopy: T_L1_ (p=0.005), T_L2_ (p=0.009), and T_L3_ (p=0.04). The percentage rise in SBP was significantly higher in the DL group during laryngoscopy at time points T_L1_ and T_L2_. The percentage rise in DBP and HR was significantly higher in the DL group at all time points during laryngoscopy. The percentage rise in DBP was also higher at certain time points post-intubation (T_6_, T_7_, T_9_, and T_10_). The percentage rise in IOP was significantly higher in the DL group post-intubation at most time points.

Conclusions: In elective neurosurgical patients, the C-MAC VL resulted in a lower rise in hemodynamic parameters and IOP as compared to DL.

## Introduction

Laryngoscopy and intubation are known to produce pressor response leading to a transient rise in heart rate (HR), mean arterial pressure (MAP), intraocular pressure (IOP), and intracranial pressure (ICP). Rise in MAP and ICP impacts cerebral perfusion in patients with intracranial space-occupying lesions undergoing neurosurgery [1-3]. The magnitude and duration of pressor response depend on the magnitude of the force used while performing laryngoscopy which is dependent on the type of laryngoscope blade and duration of laryngoscopy [4-6]. Not many studies have compared pressor responses between video laryngoscope (VL) and direct laryngoscope (DL) in the neurosurgical population. A study comparing hemodynamic responses between DL and Pentax VL (Pentax Industries, Veronella, Italy) using non-invasive blood pressure (NIBP) was done in neurosurgical patients that showed better hemodynamic stability with VL [7]. Similarly, many other studies in other surgical populations, comparing hemodynamic responses between different types of VL and DL, have used non-invasive measurement of blood pressure with hemodynamic parameters measured before and after intubation at intervals ranging from one to 10 minutes [8-16]. Invasive arterial blood pressure (IABP) provides higher accuracy and precision and allows for a higher frequency of data collection time points [17]. Studies using IABP for measuring hemodynamic responses to laryngoscopy and intubation with C-MAC® VL (C-MAC®, Karl Storz, Tuttlingen, Germany) have mostly been performed in the cardiac population [18].

This study was conducted to compare changes in invasive blood pressures, HR, and IOP consequent to laryngoscopy and intubation using a Macintosh DL (Medisearch Systems Pvt. Ltd., Jalandhar, India) and C-MAC® VL in patients undergoing brain tumor excision surgery. With the use of IABP monitoring, hemodynamic parameters were measured before laryngoscopy, while performing laryngoscopy and intubation, and at 30-second intervals post-intubation.

## Materials and methods

**Figure 1:**
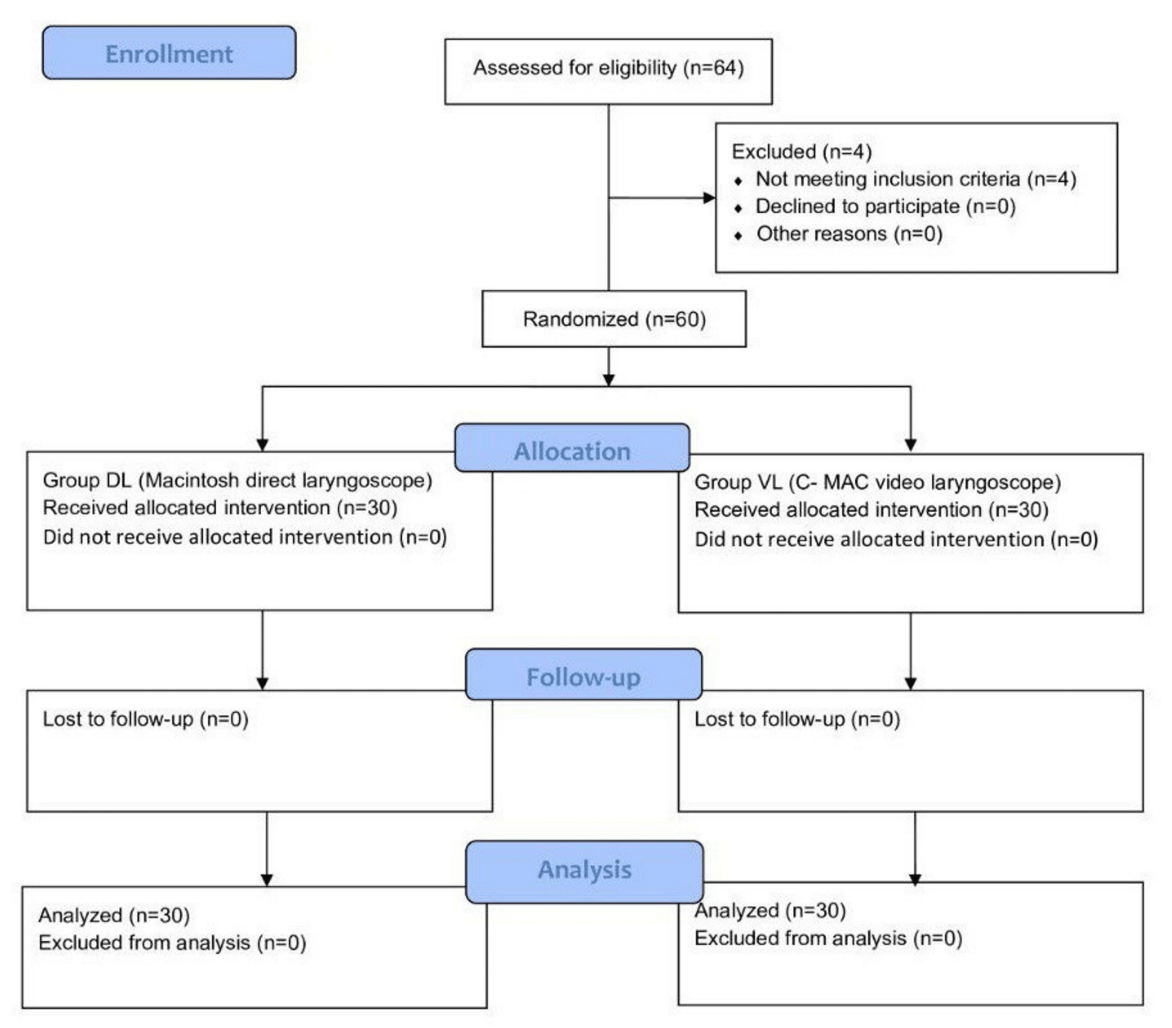
CONSORT diagram CONSORT: Consolidated Standards of Reporting Trials

This prospective interventional randomized comparative study was conducted between September 2022 and October 2023 at Vardhman Mahavir Medical College and Safdarjung Hospital, New Delhi, India, after seeking clearance from the institute's Institutional Ethics Committee (approval number: IEC/VMMC/SJH/Thesis/06/2022/CC-33). Written informed consent was obtained from all enrolled patients. The procedures followed the guidelines laid down in the 2013 Declaration of Helsinki. The trial was registered with Clinical Trials Registry-India (CTRI) (clinical trial number: CTRI/2023/03/050255) and adheres to the applicable Consolidated Standards of Reporting Trials (CONSORT) guidelines (Figure 1). Lee [8] observed that mean blood pressure after intubation was 115.2±20.4 mmHg and 135.1±22.3 mmHg in group P (Pentax airway scope (AWS) (Pentax, Tokyo, Japan)) and group M (Macintosh laryngoscope), respectively. Taking these values as reference, the minimum required sample size with a 90% study power and a 5% significance level was 25 patients in each study group. Taking a loss to analysis as 15%, the total sample size taken was 60 (30 patients per group). Patients with fronto-parietal brain tumors and having a Glasgow Coma Scale score of 15, aged 18-65 years, of either gender, belonging to American Society of Anesthesiologists (ASA) grade I or II, and scheduled for elective brain tumor excision surgery were included in the study. Patients with signs of raised ICP (clinical or radiological); anticipated difficult airway as assessed using the parameters Modified Mallampati Classes 3 and 4, inter-incisor gap <4 cm, thyromental distance <6 cm, sternomental distance <12 cm, and restricted neck movements; presence of eye disease; drug allergy; and body mass index (BMI) >30 kg m^-2^ were excluded. Block randomization in a series of 10 blocks was done to allocate patients to two groups based on the sealed envelope method. Patients were randomly allocated to two groups of 30 each: group DL was done using a Macintosh laryngoscope, and group VL was done using C-MAC® VL. The primary outcome was to compare percentage change in MAP, and secondary outcomes were to compare percentage changes in HR, systolic blood pressure (SBP), diastolic blood pressure (DBP), and IOP consequent to laryngoscopy and intubation using a Macintosh DL and C-MAC® VL. 

Patients observed fasting for six hours prior to surgery. In the operating theater, an electrocardiogram, NIBP cuff, pulse oximeter, electrodes for bispectral index (BIS), and neuromuscular monitoring for train-of-four (TOF) response were attached. Injection fentanyl (1 mcg kg^-1^, i.v.) was administered. After the local infiltration of skin and subcutaneous tissue with 2% lignocaine, a 22G arterial line (Vygon arterial leadercath polyethylene, Vygon, Ecouen, France) was inserted in the radial artery for real-time invasive blood pressure monitoring. Baseline readings of HR, IABP (SBP, DBP, MAP), and IOP were recorded five minutes after the insertion of the radial arterial line. Baseline IOP measurement was performed using the Schiotz tonometer after the administration of paracaine 0.5% drops and moxifloxacin 0.5% eye drops to both eyes, observing aseptic precautions. Three measurements were taken, and the average of the two closest readings was used for analysis. All IOP measurements were performed in the supine position with the head in neutral position.

After pre-oxygenation to obtain a 90% end tidal concentration of oxygen, induction of general anesthesia was done using injection fentanyl (1 mcg kg^-1^, i.v.) and injection thiopentone (5 mg kg^-1^, i.v.) followed by injection vecuronium bromide (0.1 mg kg^-1^, i.v.). Positive pressure mask ventilation was done for three minutes with oxygen (fraction of inspired oxygen (FiO_2_)=0.5), nitrous oxide, and isoflurane maintaining a minimum alveolar concentration (MAC) of 1. End-tidal carbon dioxide (EtCO_2_) was maintained between 30 and 35 mmHg. After achieving a TOF count of 0, laryngoscopy was performed with the head in sniffing position using either a Macintosh DL (DL group) or C-MAC® VL (VL group). The percentage of glottic opening (POGO) score and Cormack-Lehane (CL) grade were noted. Endotracheal intubation was performed using standard, curved, polyvinylchloride tubes with an 8 mm internal diameter for males and a 7 mm internal diameter for females. A styleted endotracheal tube was used in the VL group, and the stylet was removed once the tip of the tube passed the glottis. Laryngoscope was removed, and the cuff of the endotracheal tube was inflated to maintain a cuff pressure of 25-30 cm H_2_O. The endotracheal tube was fixed at a depth of 21 cm in males and 19 cm in females at the level of the upper incisors. Intubation was confirmed by the appearance of a square wave capnography trace and five-point auscultation. The procedure was done by an anesthesiologist who had performed more than 50 successful intubations with each laryngoscope, and BIS was maintained at 50-60 throughout. Volume control ventilation with a tidal volume of 5-7 ml/kg was initiated using an FiO_2_ of 0.5 with nitrous oxide and isoflurane (1 MAC) to maintain EtCO_2_ between 30 and 35 mmHg.

Hemodynamic parameters were recorded at baseline (T_baseline_) and one minute after induction (T_i_). Recordings were made during laryngoscopy at the time when the laryngoscope blade got inserted at the vallecula (T_L1_), five seconds after the laryngoscope blade got inserted at the vallecula (T_L2_), and immediately after the removal of the laryngoscope blade (T_L3_). Thereafter, post-intubation parameters were recorded at regular intervals of every 30 seconds (T_1_ to T_10_) for the next five minutes. IOP was recorded at T_baseline_, T_2_, T_4_, T_6_, T_8_, and T_10_. Laryngoscopy time was defined as the time from the insertion of the tip of the laryngoscope beyond the incisors till the removal of the tip of the blade from the oral cavity. Intubation time was defined as the time taken from the insertion of the blade beyond the incisors until four square wave patterns of EtCO_2_ appeared on the monitor. Use of additional maneuvers to aid intubation such as optimum external laryngeal manipulation or a device (bougie) to aid intubation was recorded. Each introduction and removal of the laryngoscope blade was counted as an attempt at laryngoscopy. Passage of the endotracheal tube was counted as an attempt at intubation. Any removal of the endotracheal tube and its re-insertion was counted as a subsequent attempt at intubation. The number of attempts at laryngoscopy and intubation was recorded. Occurrence of adverse events such as oxygen desaturation, bronchospasm, and arrhythmias was noted.

Categorical variables were presented in numbers and percentages (%), and continuous variables were presented as mean±SD and median. Normality of data was tested by the Kolmogorov-Smirnov test. If the normality was rejected, then a non-parametric test was used. Quantitative variables were compared using an unpaired t-test or the Mann-Whitney U test (when the data sets were not normally distributed) between the two groups. Qualitative variables were compared using the chi-squared test or Fisher's exact test, and a p-value of <0.05 was considered statistically significant. Data was entered in an MS Excel spreadsheet (Microsoft Corp., Redmond, WA, USA), and analysis was done using IBM SPSS Statistics for Windows, V. 21.0 (IBM Corp., Armonk, NY, USA).

## Results

Sixty-four patients were enrolled in the study. Four patients were excluded based on the exclusion criteria. The remaining 60 patients consented to the study and were randomly allocated into the VL group (n=30) and the DL group (n=30). The results of these 60 patients were analyzed (Figure 1). Demographic and airway examination parameters of the patients were comparable (Table 1 and Table 2). 

**Table 1:** Demographic and airway examination parameters of the study population Tests applied: ^†^chi-squared test; *Fisher's exact test; ^‡^independent t-test A p-value of >0.05 is considered non-significant. ASA: American Society of Anesthesiologists; BMI: body mass index; DL: direct laryngoscope; MMPC: Modified Mallampati Class; NA: not applicable; VL: video laryngoscope

		DL group	VL group	P-value
Age (years)	19-30	10 (33.33%)	11 (36.67%)	0.84^†^
	31-40	9 (30%)	8 (26.67%)	
	41-50	5 (16.67%)	7 (23.33%)	
	51-60	6 (20%)	4 (13.33%)	
Gender	Female	11 (36.67%)	15 (50%)	0.297^†^
	Male	19 (63.33%)	15 (50%)	
BMI (kg m^-2^)	18.5-22.99	12 (40%)	8 (26.67%)	0.165^† ​​​​​​​^
	23-24.99	7 (23.33%)	14 (46.67%)	
	>25	11 (36.67%)	8 (26.67%)	
ASA grade	I	29 (96.67%)	18 (60%)	0.26^*^
	II	1 (3.33%)	12 (40%)	
Dentition: firm		30 (100%)	30 (100%)	NA
MMPC	I	10 (33.33%)	13 (43.33%)	0.877^†^
	II	20 (66.67%)	17 (56.67%)	
Jaw protrusion: normal		30 (100%)	30 (100%)	NA
Neck movements: normal		30 (100%)	30 (100%)	NA
Inter-incisor distance (cm) mean±SD		5.16±0.44	5.02±0.37	0.177^‡^
Thyromental distance (cm) mean±SD		7.45±0.62	7.34±0.58	0.467^‡^
Sternomental distance (cm) mean±SD		14.34±0.87	14.14±1.15	0.45^‡^

**Table 2:** Laryngoscopy and intubation parameters of the study population Tests applied: ^‡^independent t-test; *Fisher's exact test A p-value of <0.05 is considered significant, while a p-value of <0.001 is considered highly significant. CL: Cormack-Lehane; DL: direct laryngoscope; POGO: percentage of glottic opening; VL: video laryngoscope

		DL group	VL group	P-value
Laryngoscopy time (seconds) mean±SD		9.17±1.32	8.37±1.07	0.012^‡^
Range		7-11	6-10	
Intubation time (seconds) mean±SD		16.03±1.81	13.7±1.09	<0.0001^‡^
Range		12-19	12-16	
CL grade	I	12 (40%)	11 (36.67%)	1^*^
	IIa	14 (46.67%)	15 (50%)	
	IIb	4 (13.33%)	4 (13.33%)	
POGO score mean±SD		86.53±7.56	86.67±7.69	0.946^‡^

Mean values of laryngoscopy time and intubation time were significantly shorter in the VL group than in the DL group (Table 2). The tracheas of all patients in both groups were intubated after a single attempt of laryngoscopy and intubation. The CL grade and POGO score of patients in both groups were comparable (Table 2). No adverse events were noted in any patient in either group. The baseline values of MAP, HR, SBP, DBP, and IOP were comparable between the two groups (Table 3, Table 4, and Table 5). The percentage change in these parameters at other time points was calculated with respect to their baseline value.

**Table 3:** MAP and HR variation due to laryngoscopy and intubation Tests applied: ^‡^independent t-test; ^§^Mann-Whitney U test Data expressed as mean±standard deviation. A p-value of <0.05 is considered significant, while a p-value of <0.001 is considered highly significant. T_baseline_: five minutes after radial arterial line insertion; T_i_: one minute after induction; T_L1_: at the time when the laryngoscope blade got inserted at the vallecula; T_L2_: five seconds after the laryngoscope blade got inserted at the vallecula; T_L3_: immediately after the removal of the laryngoscope blade; T_1_: 30 seconds post-intubation; T_2_: one minute post-intubation; T_3_: one minute and 30 seconds post-intubation; T_4_: two minutes post-intubation; T_5_: two minutes and 30 seconds post-intubation; T_6_: three minutes post-intubation; T_7_: three minutes and 30 seconds post-intubation; T_8_: four minutes post-intubation; T_9_: four minutes and 30 seconds post-intubation; T_10_: five minutes post-intubation; DL: direct laryngoscope; HR: heart rate; MAP: mean arterial pressure; VL: video laryngoscope

Time point	MAP (mmHg)			HR (beats per minute)		
	DL group	VL group	P-value	DL group	VL group	P-value
T_baseline_	89.4±9.76	88.89±9.35	0.837^‡^	81.57±11.6	79.73±13.42	0.574^‡^
T_i_	87.23±9.62	87.01±9.38	0.580^‡^	80±10.58	78.27±13.39	0.928^‡^
% change	-2.42±1.74	-2.13±1.13	0.101^§^	-1.71±3.33	-1.85±1.99	0.813^§^
T_L1_	93.4±9.86	90.92±9.08	0.315^‡^	87.8±10.66	82.1±13.34	0.073^‡^
% change	4.57±3.41	2.37±2.12	0.005^§^	8.23±7.74	3.08±2.04	0.0002^§^
T_L2_	97.5±10.9	94.67±9.02	0.277^‡^	91.77±10.88	85.63±13.46	0.057^‡^
% change	9.11±4.17	6.64±2.54	0.009^§^	13.18±9.03	7.62±3.1	0.0007^§^
T_L3_	100.1±10.92	97.27±9.4	0.286^‡^	94.33±11.2	88.03±13.84	0.057^‡^
% change	12.1±5.69	9.55±2.56	0.04^§^	16.58±11.68	10.63±3	0.003^§^
T_1_	98.93±10.82	98.21±9.22	0.782^‡^	95.2±10.77	89.6±13.41	0.08^‡^
% change	10.75±4.34	10.66±3.15	0.923^§^	18.21±17.11	12.75±3.39	0.311^§^
T_2_	97.9±10.79	98.43±9.07	0.837^‡^	94.63±10.5	89.7±13.1	0.113^‡^
% change	9.56±3.14	10.94±3.31	0.169^§^	17.48±16.43	12.96±3.88	0.408^§^
T_3_	97.2±10.97	96.27±8.87	0.718^‡^	93.23±9.73	89.47±12.95	0.208^‡^
% change	8.79±4.36	8.51±3.62	0.988^§^	15.91±17.02	12.68±4.24	0.894^§^
T_4_	95.4±11.85	95.93±9.25	0.847^‡^	92.43±9.99	87.83±12.56	0.122^‡^
% change	6.64±4.57	8.06±2.76	0.169^§^	15.03±18.15	10.66±4.22	0.391^§^
T_5_	92.53±11.03	93.61±8.5	0.673^‡^	89.47±9.05	85.23±12.05	0.13^‡^
% change	3.52±4.63	5.5±2.79	0.315^§^	11.11±14.73	7.44±4.99	0.779^§^
T_6_	91.4±10.32	91.13±8.83	0.915^‡^	87.67±9.66	82.87±11.53	0.086^‡^
% change	2.34±5.21	2.65±2.71	0.579^§^	8.78±14.62	4.56±6.49	0.303^§^
T_7_	89.3±9.72	89.3±8.81	1^‡^	85.33±10.38	81.17±11.73	0.151^‡^
% change	-0.02±4.01	0.57±2.58	0.336^§^	5.66±12.59	2.29±5.39	0.391^§^
T_8_	87±9.54	87.81±8.86	0.734^‡^	83.17±9.71	78.83±11.9	0.128^‡^
% change	-2.58±4.28	-1.15±1.78	0.092^§^	2.9±11.49	-0.74±5.77	0.115^§^
T_9_	86.87±9.28	86.88±8.91	0.996^‡^	81.6±9.29	77.53±11.86	0.145^‡^
% change	-2.71±4.17	-2.22±2.01	0.767^§^	0.83±9.24	-2.42±5.15	0.056^§^
T_10_	85.8±9.41	86.19±9.04	0.871^‡^	80.8±10.01	76.83±11.94	0.169^‡^
% change	-3.9±4.79	-3±2.46	0.848^§^	-0.28±8.56	-3.36±4.26	0.086^§^

**Table 4:** SBP and DBP variation due to laryngoscopy and intubation Tests applied: ^‡^independent t-test; ^§^Mann-Whitney U test Data expressed as mean±standard deviation. A p-value of <0.05 is considered significant, while a p-value of <0.001 is considered highly significant. T_baseline_: five minutes after radial arterial line insertion; T_i_: one minute after induction; T_L1_: at the time when the laryngoscope blade got inserted at the vallecula; T_L2_: five seconds after the laryngoscope blade got inserted at the vallecula; T_L3_: immediately after the removal of the laryngoscope blade; T_1_: 30 seconds post-intubation; T_2_: one minute post-intubation; T_3_: one minute and 30 seconds post-intubation; T_4_: two minutes post-intubation; T_5_: two minutes and 30 seconds post-intubation; T_6_: three minutes post-intubation; T_7_: three minutes and 30 seconds post-intubation; T_8_: four minutes post-intubation; T_9_: four minutes and 30 seconds post-intubation; T_10_: five minutes post-intubation; DBP: diastolic blood pressure; DL: direct laryngoscope; SBP: systolic blood pressure; VL: video laryngoscope

Time point	SBP (mmHg)			DBP (mmHg)		
	DL group	VL group	P-value	DL group	VL group	P-value
T_baseline_	121.77±12.85	120.13±11.2	0.602^‡^	74.13±10.16	73.27±9.9	0.739^‡^
T_i_	119.57±12.41	116.97±10.63	0.387^‡^	73.2±10.03	71.83±9.75	0.595^‡^
%change	-1.75±2.33	-2.61±1.14	0.315^§^	-1.18±3.84	-1.95±1.81	0.322^§^
T_L1_	124.87±15.15	122.43±10.73	0.476^‡^	77.77±10.49	75.17±9.68	0.322^‡^
%change	2.74±8.32	1.98±1.83	0.018^§^	5.07±5.1	2.72±3.06	0.008^§^
T_L2_	130.2±12.93	125.93±10.51	0.166^‡^	82.47±10.42	79.03±9.61	0.19^‡^
%change	7.06±3.71	4.94±2.35	0.003^§^	11.58±6.14	8.1±3.51	0.003^§^
T_L3_	132.37±9.87	128.93±10.27	0.192^‡^	85.63±11.54	81.43±10.26	0.142^‡^
%change	9.2±6.75	7.48±2.61	0.249^§^	15.79±7.94	11.33±3.68	0.004^§^
T_1_	130.93±11.29	130.23±10.17	0.802^‡^	85.27±10.43	82.2±10.11	0.252^‡^
%change	7.84±5.04	8.59±2.99	0.706^§^	15.4±6	12.43±4.21	0.08^§^
T_2_	128.57±12.86	129.97±10.2	0.642^‡^	84.3±10.19	82.67±10.06	0. 535^‡^
%change	5.77±5.48	8.37±3.45	0.055^§^	14.05±4.8	13.11±4.41	0.584^§^
T_3_	128.5±12.99	129.8±10.45	0.671^‡^	82.93±10.2	81.8±9.81	0.662^‡^
%change	5.69±5.67	8.22±3.52	0.051^§^	12.27±6.8	11.96±4.86	0.971^§^
T_4_	126.93±12.61	127±10.69	0.982^‡^	82.7±10.2	80.4±9.91	0.379^‡^
%change	4.47±6.3	5.84±2.82	0.122^§^	12±7.39	9.99±4.55	0.183^§^
T_5_	125.4±12.64	124.37±10.21	0.729^‡^	79.87±10.03	78.23±9	0.509^‡^
%change	3.16±5.63	3.68±3.28	0.496§	8.25±8.41	7.12±4.32	0.379^§^
T_6_	122.5±12.08	122.07±10.35	0.882^‡^	78.43±9.49	75.67±9.43	0.262^‡^
%change	0.78±5.17	1.73±2.92	0.243^§^	6.45±9.23	3.49±4.45	0.021^§^
T_7_	120.03±12.34	120.17±10.19	0.964^‡^	76.4±9.68	73.87±9.48	0.31^‡^
%change	-1.22±6.08	0.16±3.2	0.195^§^	3.72±9.91	0.97±3.83	0.03^§^
T_8_	120.97±12.08	118.1±10.34	0.327^‡^	75.03±9.1	72.67±9.47	0.328^‡^
%change	-0.48±5.22	-1.6±2.97	0.158^§^	1.79±8.53	-0.72±3.13	0.102^§^
T_9_	120.07±11.87	117.23±10.46	0.331^‡^	74.3±8.25	71.7±9.21	0.254^‡^
%change	-1.24±4.51	-2.35±2.51	0.311^§^	0.83±7.31	-2.02±3.26	0.012^§^
T_10_	119.1±11.41	116.37±10.66	0.342^‡^	74.3±8.26	71.1±9.53	0.17^‡^
%change	-2.02±4.04	-3.09±2.46	0.041^§^	0.89±8.55	-2.89±3.23	0.026^§^

**Table 5:** IOP variation due to laryngoscopy and intubation Tests applied: ^‡^independent t-test; ^§^Mann-Whitney U test Data expressed as mean±standard deviation. A p-value of <0.05 is considered significant, while a p-value of <0.001 is considered highly significant. T_baseline_: five minutes after radial arterial line insertion; T_2_: one minute post-intubation; T_4_: two minutes post-intubation; T_6_: three minutes post-intubation; T_8_: four minutes post-intubation; T_10_: five minutes post-intubation; DL: direct laryngoscope; IOP: intraocular pressure; VL: video laryngoscope

Time point	IOP (mmHg) (right)			IOP (mmHg) (left)		
	DL group	VL group	P-value	DL group	VL group	P-value
T_baseline_	12.67±2.2	13.43±2.28	0.191^‡^	12.23±2.19	13.2±2.17	0.092^‡^
T_2_	15.48±2.56	14.53±2.47	0.127^‡^	14.8±2.64	14.23±2.21	0.371^‡^
%change	23.95±17.81	8.45±7.44	<0.0001^§^	22.14±16.47	8.06±4.33	<0.0001^§^
T_4_	14.93±1.98	15±2.38	0.907^‡^	14.8±1.97	14.8±2.27	1^‡^
%change	19.52±15.12	12.12±7.45	0.021^§^	22.79±16.29	12.49±6.3	0.003^§^
T_6_	13.97±2.44	14.03±2.13	0.911^‡^	13.86±2.2	13.93±1.87	0.95^‡^
%change	11.79±19.77	5.08±8.39	0.323^§^	15.83±17.3	6.43±8.98	0.013^§^
T_8_	13.43±2.13	13.57±1.96	0.802^‡^	13.2±2.23	13.3±2.22	0.862^‡^
%change	7.1±13.47	1.81±9.17	0.271^§^	9.4±17.59	1.15±8.99	0.057^§^
T_10_	12.63±2.04	13.4±2.01	0.148^‡^	12.63±2.09	12.9±2.06	0.621^‡^
%change	0.28±8.54	0.36±7.98	0.927^§^	4.2±11.49	-1.98±5.44	0.007^§^

The percentage rise in MAP from its baseline value (Table 3) was significantly higher in the DL group as compared with the VL group at all time points during laryngoscopy: T_L1_ (p=0.005), T_L2_ (p=0.009), and T_L3_ (p=0.04). It was comparable between the two groups post-intubation at all time points. The percentage rise in HR (Table 3) was significantly higher in the DL group than the VL group at all time points during laryngoscopy: T_L1_ (p=0.0002), T_L2_ (p=0.0007), and T_L3_ (p=0.003). It was comparable between the two groups post-intubation. The percentage rise in SBP (Table 4) was significantly higher in the DL group at two time points during laryngoscopy: T_L1_ (p=0.018) and T_L2_ (p=0.003). At time point T_10_, SBP fell slightly below baseline in both groups but continued to stay higher in the DL group as compared with the VL group (p=0.041). The percentage change in SBP was comparable at the remaining time points between the two groups. The percentage rise in DBP (Table 4) was significantly higher in the DL group at all time points during laryngoscopy, that is, T_L1_ (p=0.008), T_L2_ (p=0.003), and T_L3_ (p=0.004), and also post-intubation at time points T_6 _(p=0.021), T_7 _(p=0.03), T_9_ (p=0.012), and T_10_ (p=0.026). It was comparable between the two groups at the remaining time points. The percentage rise in IOP in the left eye (Table 5) from its baseline value was significantly more in the DL group at time points T_2_ (p<0.0001), T_4 _(p=0.003), T_6 _(p=0.013), and T_10 _(p=0.007). The percentage rise in IOP in the right eye (Table 5) was significantly more in the DL group at time points T_2_ (p<0.0001) and T_4_ (p=0.021). EtCO_2_ concentration was maintained between 30 and 35 mmHg in both groups, and no adverse events were recorded.

## Discussion

Reflex circulatory reactions triggered by different laryngoscopy blades for tracheal intubation under general anesthesia have been extensively studied [7-16,18]. Most studies have used NIBP monitoring, have measured parameters at intervals ranging from one to 10 minutes post-intubation, and yielded variable and conflicting results [7-16]. IABP monitoring is real time, offers higher accuracy, and allows for increased frequency of measurements [17]. It is the standard of care for monitoring hemodynamics in certain neurosurgical patients [19]. Hence, this study was planned in the neurosurgical population, and IABP was used for recording changes in blood pressure (MAP, SBP, DBP). Hemodynamic values were recorded not only at baseline, post-induction, and post-intubation at an interval of 30 seconds (T_1_-T_10_) but also while the operator performed laryngoscopy and intubation (time points T_L1_, T_L2_, T_L3_). IOP was recorded at baseline and post-intubation. 

Demographic characteristics, airway parameters, CL grade, and POGO score of the study population were comparable between the two groups (Table 1 and Table 2). The mean laryngoscopy time was 8.37±1.07 seconds in the VL group and 9.17±1.32 seconds in the DL group. The mean intubation time was 13.7±1.09 seconds in the VL group and 16.03±1.81 seconds in the DL group. The laryngoscopy time and intubation time were both statistically higher (p=0.012 and p<0.001, respectively) in the DL group as compared with the VL group (Table 2). Many studies have reported earlier glottic visualization, better laryngoscopy glottic views, but longer intubation times with C-MAC® and other types of videolaryngoscopes [8-10,20]. Improved glottic visualization along with comparable intubation time with the use of C-MAC® VL as well as other types of VL has also been reported [7,21-23]. Similar to our study, shorter laryngoscopy and intubation times with the use of certain other VL as compared with DL have also been reported [11]. In the present study, a significantly lower intubation time with the use of C-MAC® VL could probably be due to the exclusion of patients with anticipated difficult airways and intubations being performed by experienced anesthesiologists who had already performed more than 50 intubations using C-MAC® VL. All patients were intubated in a single attempt of laryngoscopy and intubation, with none of them requiring any additional use of bougie or optimal external laryngeal manipulation (OELM), thus ruling out any additional manipulation that could have led to an increased sympathetic response. 

Percentage change in MAP and DBP with respect to baseline value was significantly higher in the DL group as compared with the VL group at all time points during laryngoscopy: T_L1 _(p=0.005 and p=0.008, respectively), T_L2 _(p=0.009 and p=0.003, respectively), and T_L3_ (p=0.04 and p=0.004, respectively). Percentage change in SBP with respect to its baseline value was significantly higher in the DL group as compared with the VL group at time points T_L1_ (p=0.018) and T_L2_ (p=0.003) during laryngoscopy. 

Percentage change in DBP was also significantly higher post-intubation in the DL group when compared with the VL group at T_6_ (p=0.021), T_7_ (p=0.03), T_9_ (p=0.012), and T_10_ (p=0.026). Percentage change in heart rate with respect to baseline was significantly higher at all time points during laryngoscopy T_L1_ (p=0.0002), T_L2 _(p=0.0007), and T_L3_ (p=0.003) in the DL group as compared with the VL group. Percentage change in hemodynamic parameters was comparable at all other time points between the study groups. 

A significant rise in MAP, HR, SBP, and DBP at most time points during the post-intubation period with the Macintosh DL when compared with C-MAC® VL has been reported [12]. Similar results were obtained in the neurosurgical population with Pentax AWS [7]. Lee reported significantly higher percentage change of SBP, MAP, DBP, and HR at most time points post-intubation with Mcintosh DL as compared with Pentax video laryngoscopy, although measured serum norepinephrine levels were comparable between the two groups [8]. The time point T_0_ in their study corresponded with T_L3_ of the present study. Similar results were obtained at this time point (when the laryngoscope blade was removed from the oral cavity), which was a significantly higher percentage change in HR, DBP, and MAP with the DL group. Similar to the current study, another study was performed in which IABP was used for hemodynamic monitoring and the parameters were measured during the process of laryngoscopy as well as post-intubation. The above-mentioned study compared C-MAC® VL with another VL in the cardiac surgical population and found that the hemodynamic response with the use of C-MAC® VL was lower than the other group [18]. In the present study, real-time invasive blood pressure readings were also recorded at various steps during laryngoscopy, during intubation, and immediately following intubation.

Few studies have found a comparable rise in hemodynamics with the use of C-MAC® VL with DL [9,10,13,16]. Rarely, a significantly higher percentage change in MAP, SBP, DBP, and HR was reported with C-MAC® VL compared with Macintosh DL after intubation [14]. Use of NIBP instead of IABP and lower frequency of readings (one to five minutes) could explain the different results obtained as mentioned above. Different results in one study that also recorded hemodynamic variables during laryngoscopy and intubation could also be because of the use of glycopyrrolate premedication [13].

The values of IOP at baseline were comparable between the two groups. Post-intubation, the percentage change in IOP in the left eye was significantly more in the DL group at time points T_2_ (p<0.0001), T_4 _(p=0.003), T_6_ (p=0.013), and T_10_ (p=0.007). For the right eye, a change in IOP was found to be significantly higher in the DL group at time points T_2_ (p<0.0001) and T_4_ (p=0.021). Similarly, significantly higher IOP was reported with the use of Macintosh DL as compared with C-MAC® VL and with various other types of VL [11,12]. Sympathoadrenal stimulation caused by the distortion of supraglottic structures by the placement of the laryngoscope blade is known to raise IOP by increasing central venous pressure and increasing resistance to aqueous humor outflow [24].

Optimum visualization of the glottis using DL requires alignment of the oral, laryngeal, and pharyngeal axes and laryngeal manipulation requiring the application of higher forces. On the contrary, with the use of VL, the need for alignment of the three axes is eliminated. Laryngoscopy and intubation require lesser manipulation and application of lower forces. These manipulations and the magnitude of forces may affect the sympathetic response and its consequences such as hemodynamic alterations and a rise in IOP [4-6,25]. Also, significantly lower laryngoscopy time and intubation time with the use of C-MAC® VL in the present study could have led to lesser sympathoadrenal pressor responses. 

The main strengths of the study were that for measuring the pressor response, hemodynamics were measured at multiple time points, while performing laryngoscopy and intubation, and at an interval of every 30 seconds post-intubation. Real-time values of arterial pressure were recorded by inserting a radial arterial catheter. Measurements of IOP were also included. However, limitations were that the study was conducted in ASA grade I or II subjects with a normal airway, so the results cannot be applied to patients with a difficult airway. The operator was not blinded to the type of laryngoscope blade used. Also, ICP was not recorded directly. ICP is an invasive neuro-monitoring technique. Subjects of our study were elective neurosurgery patients with fronto-parieto-temporal lesions, with no evidence of raised ICP in whom ICP monitoring was not indicated. Further research can be done by comparing different types of VL and evaluating their role in high-risk or difficult airway populations. 

## Conclusions

Minimizing sympathoadrenal pressor response and consequential rise in arterial pressures, IOP, and ICP arising from laryngoscopy and intubation is crucial for patients undergoing neurosurgery. The C-MAC® VL may be preferred over the Macintosh DL in such patients.
